# Coordination Pattern of the Thigh, Pelvic, and Lumbar Movements during the Gait of Patients with Hip Osteoarthritis

**DOI:** 10.1155/2020/9545825

**Published:** 2020-07-24

**Authors:** Takuya Ibara, Masaya Anan, Ryosuke Karashima, Kiyotaka Hada, Koichi Shinkoda, Mahito Kawashima, Makoto Takahashi

**Affiliations:** ^1^Graduate School of Biomedical and Health Sciences, Hiroshima University, 2-3, Kasumi 1-Chome, Minami-ku, Hiroshima 734-8553, Japan; ^2^Department of Rehabilitation, Kawashima Clinic, 11-1 Miyabu, Nakatsu, Oita 871-0012, Japan; ^3^Physical Therapy Course, Faculty of Welfare and Health Science, Oita University, 700, Dannoharu, Oita 870-1192, Japan; ^4^Department of Biomechanics, Graduate School of Biomedical and Health Sciences, Hiroshima University, 2-3, Kasumi 1-Chome, Minami-ku, Hiroshima 734-8553, Japan; ^5^Center for Advanced Practice and Research of Rehabilitation, Graduate School of Biomedical and Health Sciences, Hiroshima University, 2-3, Kasumi 1-Chome, Minami-ku, Hiroshima 734-8553, Japan; ^6^Department of Orthopaedic, Kawashima Orthopaedic Hospital, 17 Miyabu, Nakatsu, Oita 871-0012, Japan

## Abstract

There are limited reports on segment movement and their coordination pattern during gait in patients with hip osteoarthritis. To avoid the excessive stress toward the hip and relevant joints, it is important to investigate the coordination pattern between these segment movements, focusing on the time series data. This study aimed to quantify the coordination pattern of lumbar, pelvic, and thigh movements during gait in patients with hip osteoarthritis and in a control group. An inertial measurement unit was used to measure the lumbar, pelvic, and thigh angular velocities during gait of 11 patients with hip osteoarthritis and 11 controls. The vector coding technique was applied, and the coupling angle and the appearance rate of coordination pattern in each direction were calculated and compared with the control group. Compared with the control group, with respect to the lumbar/pelvic segment movements, the patients with hip osteoarthritis spent more rates in anti-phase and lower rates in in-phase lateral tilt movement. With respect to the pelvic/thigh segment movements, the patients with hip osteoarthritis spent more rates within the proximal- and in-phases for lateral tilt movement. Furthermore, patients with osteoarthritis spent lower rates in the distal-phase for anterior/posterior tilt and rotational movement. Patients with hip osteoarthritis could not move their pelvic and thigh segments separately, which indicates the stiffness of the hip joint. The rotational movement and lateral tilt movements, especially, were limited, which is known as Duchenne limp. To maintain the gait ability, it seems important to pay attention to these directional movements.

## 1. Introduction

Patients with hip osteoarthritis (OA) often have altered gait patterns, which reportedly may accelerate disease progression and severity or worsen symptoms [1–3]. Altered gait patterns involving unusual movements in the hip joint cause other joints to move excessively to compensate for the hip joint. These compensational movements may put additional stress on these joints, leading to dysfunction [4, 5], represented as coxitis knee [6] or hip-spine syndrome [5, 7]. It can be assumed that the cause of these compensational movements is the hip stiffness. Steinhilber et al. [8] showed that patients with hip OA are likely to report the complaint of hip stiffness. Tateuchi et al. [9] reported that patients with hip OA who had undergone total hip replacement (THR) were likely to experience dynamic hip joint stiffness, whereas Foucher et al. [10] reported that patients were likely to retain their preoperative gait pattern after THR. Although the dynamic range of motion of the hip during gait had reportedly improved among post-THR patients, it was not comparable with that in normal individuals [10]. Other studies also examined the gait of patients with hip OA who had not undergone THR by studying joint kinematics, kinetics, and their correlations [4, 11–15]. But, it remains unclear whether the hip joint stiffness is present in the gait of patients with hip OA preoperatively. Traditional measurements, such as examining the range of motion during gait, are not enough for understanding control mechanisms. It is important to examine the gait and movement patterns of patients with hip OA by focusing on time interval such as joint coordination [16]. The vector coding technique (VCT) allows researchers to examine the coordination pattern of the movement over time [17, 18]. Several studies found that the people with pain or musculoskeletal disorders, such as patellofemoral pain, low back pain, or idiopathic scoliosis, showed less movement between two relevant segments or joints using the VCT [18–20]. Smith et al. [21] investigated the coordination pattern between pelvis and thigh with weak hip muscle in healthy subjects, and Samaan [22] studied the coordination pattern between hip and knee movement in hip OA patients with VCT technique. However, there was no study that investigated the thigh–pelvis–lumbar coordination pattern of patients with hip OA preoperatively. The VCT characterizes the movement coordination pattern of two segments or joints as the predominance of movement speed using the following four phases [16, 23]; in-phase (the same directional movement with similar movement speed), anti-phase (opposite directional movement with similar movement speed), proximal-phase (relatively large proximal joint/segment movement speed), and distal-phase (relatively large distal joint/segment movement speed). The “in-phase” movement, especially, indicates a particular movement pattern with similar movement speed between two segments or joint [16, 23]. The “in-phase” and “stiff” are not actually equal in point of considering the force applied, former referring to the observed movement only and latter referring to the movement as the response to the force applied. However, it has been conceivable that the in-phase coordination pattern relates to the stiffened condition [20]. We believe that using the VCT to directly assess the patterns of movement for patients with hip OA will give us the knowledge regarding the stiff hip movement during the pelvic/thigh “in-phase” coordination pattern, as well as the relationship between the hip and relevant region movements over time in patients with hip OA.

We measured changes in segmental movement using an inertial measurement unit (IMU). The IMU is inexpensive and easy to use in a clinical setting because the IMU does not need the laboratory setting which is needed for using motion capture system. Other groups have documented the reliability and validity of using an IMU for measuring gait kinematics [24–26] and assessing the symptoms of musculoskeletal disorders [15, 25, 27, 28]. In the first step of VCT, the change in the angles of two segmental/joint movements can be calculated. The procedure thus enables the calculation of the angular velocity, which is the difference between two successive angles. Thus, we decided to use this procedure in addition to collecting angular velocity data using IMU.

The aim of this study was to investigate the coordination pattern of segmental movements in the gait of patients with hip OA using VCT and IMU, which allows us to suggest the presence of stiffness and the relationship of each segmental movement. We hypothesized that patients with hip OA will reveal increased rate of “in-phase” movement pattern due to stiffness in the hip joint. We also hypothesized that the in-phase coordination pattern of pelvic/thigh movements would have an anterior/posterior tilt due to limited hip extension movements among patients with hip OA.

## 2. Materials and Methods

### 2.1. Participants

The present study included 11 patients with hip OA from an outpatient clinic and 11 age- and gender-matched community-dwelling control volunteers. The inclusion criteria of the hip OA group were as follows: being female, ability to walk without the aid of medical equipment, age 40–70 years, and having a grade of 3 or 4 on the Kellgren-Lawrence grading system. There were very few male patients and the presence of differences with respect to patient sex was anticipated; therefore, we did not include male patients in the study. Patients with neurological conditions and other lower extremity joint disorders which affected the required tasks were excluded from the study. Among patients with bilateral hip OA, the more affected side was chosen as the “targeted” side. The control group included volunteers who did not have any complaint about their hips. The other criteria were the same as those for hip OA group. Data on study participants are found in Table 1.

All procedures were approved by the institutional ethics committee, and all participants provided written informed consent prior to participating in this study. The experimental procedures of the study were conducted according to the Declaration of Helsinki.

### 2.2. Data Collection

Three IMUs (TSND151; ATR-promotions, Soraku, Japan), each equipped with a triaxial accelerometer, gyroscope, and magnetometer, were attached to the following areas: on the lateral side of the thigh between the femoral condyle and greater trochanter (thigh), on the dorsal side between both posterior superior iliac spines (pelvis), and on the dorsal side at the first lumbar spinous process (lumbar). Sensor axes were aligned to carefully detect the anterior-posterior, medial-lateral, and superior-inferior directions of movements. The sampling rate was set at 100 Hz.

The participants were required to perform a uniform set of tasks. First, the participants were asked to stand quietly. After standing quietly, they were directed to begin walking over a distance of 30 m following an oral cue by the examiner. Participants were asked to walk the path until the examiner gave them an oral cue to stop. Once directed to stop walking, participants were required to maintain a quiet standing posture until given an oral cue by the examiner. Participants did not wear shoes during these tasks and were asked to maintain a comfortable gait speed while performing the tasks.

### 2.3. Data Analysis

The angular velocity and acceleration rates of each participant's gait were recorded as they completed the task. The initial and final moments of contact for the target limb were identified using the recorded pelvis vertical acceleration data according to a method found in a previous report [29]. The thigh anterior/posterior tilt angular velocity data was used to distinguish between the left and right initial contact. Except for the first two gait cycles, fifteen successive stance phases of gait cycles were further analyzed for this study.

The time of each stance phase was normalized to a 100-point data scale as reported previously [17]. To distinguish between the lumbar/pelvic and pelvic/thigh coordination patterns, the coupling angle (CA) was calculated using the directional angular velocities of each segment. In this study, the thigh external rotation, posterior tilt (flexion), and lateral tilt (adduction) were considered positive values. Regarding pelvic and lumbar movements, rotation to ipsilateral side of target limb, posterior tilt, and lateral obliquity toward the ipsilateral side of the target limb were considered positive values.

In the original report [17], the CA was calculated using the arctangents of the differences in angle data during successive two instants of two segments. Because the difference in angle data at successive two instants is the angular velocity vector, we used the angular velocity data recorded from IMU to calculate CA as follows:

(1) $$\begin{array}{c} \gamma_{i}=\;\tan^{-1}\left(\frac{\alpha_{i}}{\beta_{i}}\right), \end{array}$$

where *γ* was the CA calculated from the distal segment angular velocity (*α*) and the proximal segment angular velocity (*β*) at time *i*.

The calculated CA at each time point was then averaged over the 15 stance phases. For calculating average CA, the CA vectors were divided into horizontal $\left(\bar{x}\right)$ and vertical components $\left(\bar{y}\right)$, and then the averaged CA $\left(\bar{\gamma }\right)$ was calculated. This procedure was adjusted within subject as follows:

(2) $$\begin{array}{c} \bar{x_{i}}=\frac{1}{n}\sum_{i=1}^{n}\left(\cos\gamma_{i}\right), \end{array}$$

(3) $$\begin{array}{c} \bar{y_{i}}=\frac{1}{n}\sum_{i=1}^{n}\left(\sin\gamma_{i}\right), \end{array}$$

(4) $$\begin{array}{c} \bar{\gamma_{i}}=\left\{\begin{array}{cc} \arctan\left(\frac{\bar{y_{i}}}{\bar{x_{i}}}\right), & \bar{x_{i}}>0,\\ 180+\arctan\left(\frac{\bar{y_{i}}}{\bar{x_{i}}}\right), & \bar{x_{i}}>0. \end{array}\right. \end{array}$$

**Figure 1:**
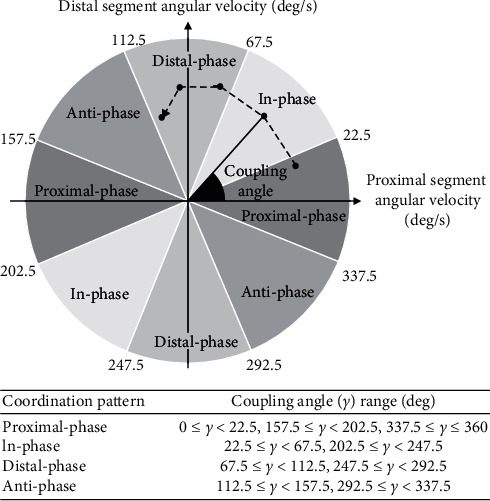
The coupling angle between the resultant vector of proximal and distal segments, angular velocity data, and the right horizontal line. The coupling angle was divided into four coordination patterns.

The averaged CA within subjects was classified at each instant according to the following four conditions: proximal-phase, in-phase, distal-phase, and anti-phase [16, 23] (Figure 1). It was expected that the “in-phase” pattern was associated with the presence of stiffness, because the proximal and distal segments tend to move together. The appearance rates, that is, the occupancy rate of the target phase during stance phase, of classified coordination patterns during the averaged CA were used for statistical analysis. To determine the times series data for the typical CA within each group, we also calculated the averaged CA within groups using the same procedure described above. Furthermore, we calculated the coupling angle variability (CAV) as the index of variability [16], which was calculated by the length of averaged coupling angle $\left(\bar{\delta_{i}}\right)$ as follows:

(5) $$\begin{array}{c} \bar{\delta_{i}}=\sqrt{{\bar{x_{i}}}^{2}+{\bar{y_{i}}}^{2}}, \end{array}$$

(6) $$\begin{array}{c} {\text{CAV}}_{i}=\sqrt{2\cdot \left(1-\bar{\delta_{i}}\right)}\cdot \frac{180}{\pi }. \end{array}$$

Additionally, we calculated the maximum value of segment angular velocity in each direction of each segment during stance phase by calculating the average of the maximum values in each stance phase.

Data Analysis was performed with using MATLAB 2017a (MathWorks, Natick, MA, USA).

### 2.4. Statistical Analysis

The anthropometric data was compared with independent-sample *t*-test, Welch's *t*-test, or Mann–Whitney *U* test in accordance with the normality (Shapiro–Wilk test) and homoscedasticity of the data. The appearance rates for coordination patterns and the angular velocity data between the control and hip OA groups were compared with using the same process as the anthropometric data except for the use of the Bonferroni correction. For the anthropometric data and the angular velocity data, the effect size was calculated using Cohen's d. Because the body weight and the body mass index were significantly different between the control group and the hip OA group, we also performed the analysis of covariance (ANCOVA) with body mass index as covariate and calculated *η*^2^ as the effect size. We checked for group by body mass index interaction for each parameter, since adjustments were meaningful only if regression slopes were homogeneous. As a result, we performed ANCOVA only for the parameters for which we found no evidence of interaction, angular velocity of thigh lateral tilt, and lumbar contralateral rotation. The significance level was set at 5%. Statistical analyses were performed using SPSS 17.0 J for Windows (SPSS Japan, Tokyo, Japan).

## 3. Results

### 3.1. Coordination Patterns

**Figure 2:**
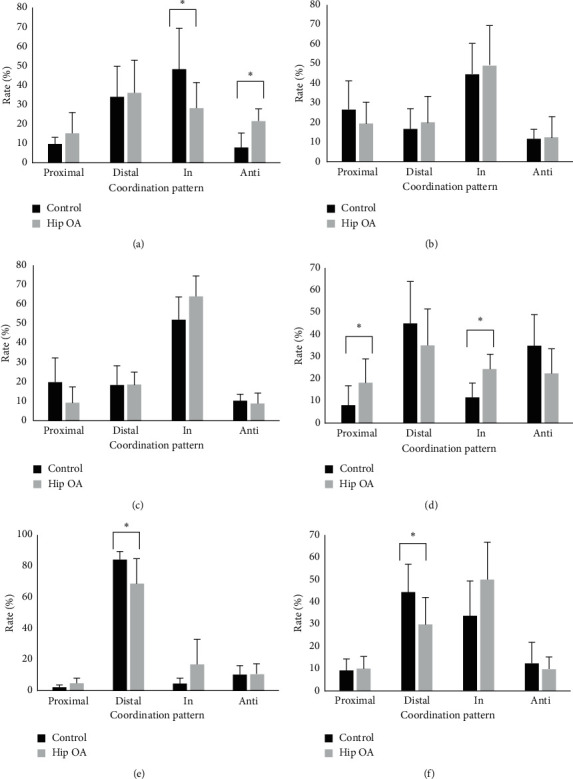
The appearance rate of each phase at the lumbar/pelvic and pelvic/thigh segments about each directional movement. The rate of appearance of lumbar/pelvic regions about lateral tilt (a), anterior/posterior tilt (b), and rotation movement (c) and that of pelvic/thigh regions about lateral tilt (d), anterior/posterior tilt (e), and rotation movement (f). ^∗^: *p* < 0.05.

Figure 2 shows the appearance rates of classified coordination pattern of lumbar/pelvic and pelvic/thigh of both groups. Regarding the lateral tilt movement of the lumbar/pelvic region (Figure 2(a)), there were significant differences between the in-phase and anti-phase appearance rates. Patients in the hip OA group spent more rates in anti-phase and lower rates in in-phase compared with control group. There were no significant differences in anterior/posterior tilt and rotational movement at lumbar/pelvic region (Figures 2(b) and 2(c)).

With regard to the pelvic/thigh, there were significant differences between the OA and control groups regarding the proximal-phase and in-phase appearance rates for lateral tilt movement (Figure 2(d)), distal-phase rate for anterior/posterior tilt movement (Figure 2(e)), and distal-phase rate for rotational movement (Figure 2(f)). Participants in the hip OA group spent more rates in the proximal- and in-phases for lateral tilt movement and lower rates in the distal-phase rate for anterior/posterior tilt and rotational movement compared with participants in the control group.

### 3.2. Time Series Data of CA and CAV

**Figure 3:**
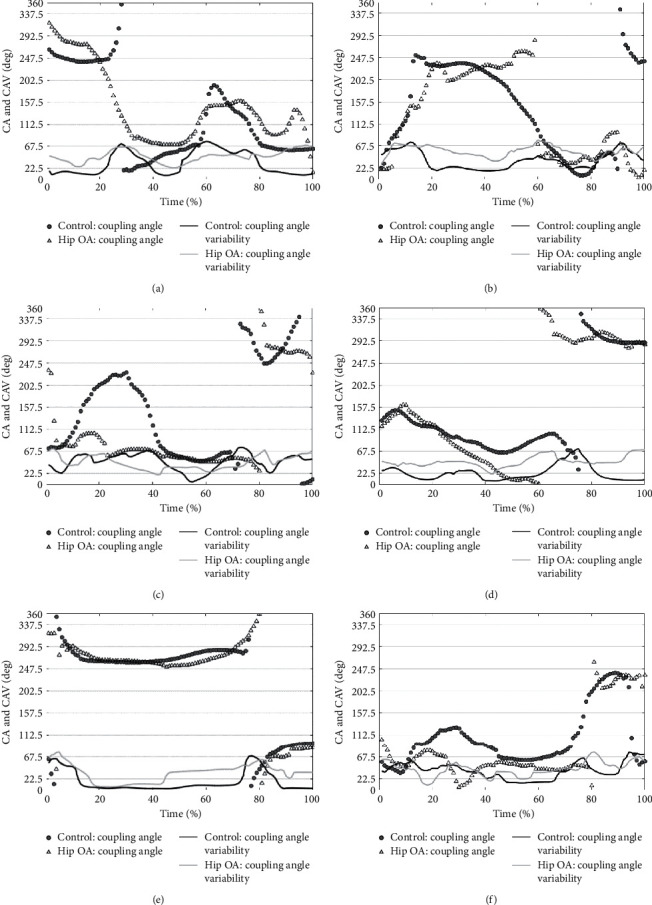
The time series data showing the mean coupling angle and coupling angle variability at lumbar/pelvic and pelvic/thigh segments about each directional movement. The mean coupling angle and coupling angle variability at lumbar/pelvic regions about lateral tilt (a), anterior/posterior tilt (b), and rotation movement, (c) and that at the pelvic/thigh regions about lateral tilt (d), anterior/posterior tilt (e), and rotation movement (f). The gray circle indicates the mean coupling angle of the control group. The white triangle indicates the mean coupling angle of the hip OA group. The gray line indicates the coupling angle variability within the hip OA group. The black line indicates the coupling angle variability within the control group.

The CA and CAV measurements for the control and hip OA groups are found in Figure 3. In regard to the lateral tilt movements of the lumbar/pelvic segments among patients with OA (Figure 3(a)), there was considerable increase in anti-phase and significant decrease in the time of in-phase movements after the initial contact of stance phase (0%–20%). The CA about the anterior/posterior tilt (Figure 3(b)) of the OA group did not decrease during the middle third of the stance phase (40%–60%). Furthermore, the CA about rotation movement remained constant during the first half of the stance phase (20%–40%) (Figure 3(c)).

Among patients with OA (Figure 3(d)), there was a considerable increase during time spent in in-phase and proximal-phase pelvic/thigh movements about the lateral tilt during middle one-third of the stance phase (30%–60%). There was a considerable decrease in the distal-phase movement about the anterior/posterior tilt (Figure 3(e)) during 70%–80% of the stance phase, and the decrease in the distal-phase movement about rotation (Figure 3(f)) was shown at 40%–80% of stance phase. The CAV was higher in the hip OA group compared with control group, except for the lumbar/pelvic and pelvic/thigh rotational movements (Figures 3(c)–3(f)).

### 3.3. Maximal Angular Velocity

There were significant differences between the control and OA groups regarding thigh angular velocities about the medial tilt, anterior tilt, and posterior tilt (Table 2). The hip OA group showed lower angular velocity in these movements compared with the control group.

## 4. Discussion

In this study, the coordination patterns of the segmental groups were assessed using the CA, which was calculated using the angular velocity data recorded using the IMU from two segments during gait.

We hypothesized that the hip OA patients would spend a lot of rate in in-phase movement pattern particularly in anterior/posterior tilt movement at pelvic/thigh segments. However, there was no significant difference in appearance rate of in-phase between both groups, although it tended to be high in hip OA group compared with control group. Furthermore, the distal-phase appearance rate was significantly lower in the OA group compared with control group. These findings may imply that the patients with hip OA relied on pelvic anterior tilt rather than hip extension when producing the stride length as described in a previous study [4], and this may be caused by the hip joint stiffness as previously reported [9, 13]. Moreover, this may be the cause of degenerative spondylolisthesis [30, 31]. Actually, three out of 11 patients of the present hip OA group were diagnosed with degenerative spondylolisthesis. This finding may also be confirmed by the relationship between the smaller maximal angular velocity value of the thigh and insignificant difference about maximal angular velocity of the pelvis anterior/posterior tilt among patients in the hip OA group.

Regarding lateral tilt movement, the OA group spent a high rate in the in-phase and proximal-phase for pelvic/thigh segment movements. By contrast, they spent a low rate in-phase and high rate in the anti-phase for lumbar/pelvic movements. These coordination patterns were observed within the first half of the stance phase, with difference in lumbar/pelvic coordination preceding the difference in the pelvic/thigh coordination pattern (Figures 3(a) and 3(d)). It was previously thought that these patterns were a symptom of the Duchenne limp, which was frequently seen in patients with hip OA [15, 32]. The Duchenne limp is a movement performed in order to compensate for weakened hip abductor muscles [15, 32]. In this study, it was demonstrated that this movement pattern preceded the body weight-bearing increment (single support phase). Furthermore, this finding means that the patients in the hip OA group may adjust their movement pattern when anticipating the successive load increment to the hip joint or abductor muscles. This finding could not recognize the maximal angular velocity because the data did not include time interval.

In rotational movement, a significant difference in movement was only observed in the distal-phase appearance rate of the pelvic/thigh region (Figure 2(f)), and the CA of the hip OA group was low during the last half of the stance phase (Figure 3(f)). In this movement, the in-phase appearance rate was not significantly different, but the difference was large in magnitude. These data may imply that individuals in the hip OA group were unable to move their pelvic and thigh segments independently from each other. One possible reason behind this stems from the instability caused from a decrease in femoral head coverage and/or the demand to make the stride length not only the extension movement, but also the rotation. The result of a previous study [33] showed that the anterior femoral head coverage decreased during late stance. Most previous reports [13, 14] showed that the range in hip extension movement among patients with hip OA decreased during the late stance. Patients compensated for the decrease in coverage of the anterior femoral head via the anterior pelvic tilt [34], which led to a decrease in the hip extension range. The hip external rotation (femoral external rotation relative to the pelvis) movement may also decrease the anterior coverage of the femoral head during late stance. Thus, the decrease of the appearance rate of separated rotational movement at the pelvic/thigh segments indicates that the patients with hip OA fix their acetabular roof toward the femoral head by making their hip joint stiff.

Throughout all the directional movements in this study, the patients with hip OA seemed to move in “in-phase” movement pattern within their hips preoperatively by making their hip stiff by their own or by the contracture. These findings can be obtained by examining the time course of joint coordination pattern during gait using VCT instead of the conventional measurements, such as the range of motion or angular velocity of each joint. With respect to lumbar/pelvic coordination, the patients with hip OA moved their pelvis compensatory especially in lateral tilt movement. The movements in anterior/posterior tilt and rotation may compensate within other segments/joints such as thorax.

This study had several limitations. First, the sample size was relatively small compared with previous studies. Previous reports suggested that not all patients of hip OA had the Duchenne limp, and there were variations within the patient pool [15]. Secondly, the symptoms of OA were relatively severe among the patients who participated in this study. Because the movement coordination patterns may be influenced by the range of motion in the hip, a larger sample size and further subdivisions of groups may be necessary. Moreover, thoracic movement was not measured in this study. The lumbar segment movement implies thoracic movement, especially in rotation and lateral tilt, because of their anatomical structures. But it remains unclear. Finally, the angular velocity data collected in this study was not measured using a global coordinate system, only a local coordinate system. Therefore, it is important to take this into account when comparing the results of this study with results collected using a motion capture system. Although there were some limitations in this study, the information collected regarding the coordination pattern of segment movement will be helpful for improving physical therapy approaches for patients with hip OA.

## 5. Conclusion

This study investigated the coordination pattern of lumbar, pelvis, and thigh movements among patients with hip OA. These patients showed less separated pelvic/thigh movements compared with control participants suggesting stiffness of the hip joint. However, patients with OA also moved their lumbar/pelvic regions in opposite directions in lateral tilt and in the same directions in rotational movement. These movements may decrease stress on the abductor muscles and provide stability to the hip joint.

## Figures and Tables

**Table 1:** Anthropometric data of control and hip OA groups.

Parameter	Control group	Hip OA group	*p*	Effect size
Age (year)	53.8 (6.9)	58.1 (7.4)	0.178	0.600
Body mass (kg)	49.3 (5.1)	59.5 (8.3)	0.002	1.490
Body height (m)	1.57 (0.05)	1.58 (0.06)	0.583	0.240
Body mass index (kg/m^2^)	20.1 (2.6)	23.8 (2.7)	0.004	1.380
OA grade (III/IV)	—	5/6	—	—

Data are represented as means (SD).

**Table 2:** The maximal angular velocity data of each segment of control and hip OA groups.

Segments	Direction	Control	Hip OA	*p*	Effect size
Lumbar	Ipsilateral tilt (deg/s)	17.8 (14.0–29.4)	15.4 (14.0–17.6)	0.200	0.364
	Contralateral tilt (deg/s)	18.1 (5.9)	14.9 (3.3)	0.129	0.249
	Anterior tilt (deg/s)	21.6 (7.6)	20.7 (7.3)	0.781	0.063
	Posterior tilt (deg/s)	30.4 (10.7)	26.2 (9.2)	0.341	0.272
	Ipsilateral rotation (deg/s)	41.1 (11.0)	40.9 (9.4)	0.955	0.016
	Contralateral rotation (deg/s)	37.5 (10.0)	29.6 (12.2)	0.841	0.002

Pelvis	Ipsilateral tilt (deg/s)	35.1 (8.5)	26.7 (11.7)	0.069	0.545
	Contralateral tilt (deg/s)	33.9 (31.7–35.9)	24.7 (20.8–34.5)	0.071	0.527
	Anterior tilt (deg/s)	22.2 (7.6)	22.7 (8.6)	0.885	0.035
	Posterior tilt (deg/s)	23.5 (7.0)	23.5 (9.9)	0.996	0.001
	Ipsilateral rotation (deg/s)	41.9 (8.9)	45.2 (18.0)	0.587	0.198
	Contralateral rotation (deg/s)	38.5 (10.5)	34.6 (19.2)	0.565	0.219

Thigh	Lateral tilt (deg/s)	53.7 (17.3)	41.1 (18.2)	0.952	0.001
	Medial tilt (deg/s)	90.5 (25.0)	49.8 (21.4)	**0.001**	1.944
	Anterior tilt (deg/s)	108.9 (25.4)	82.4 (22.5)	**0.017**	1.252
	Posterior tilt (deg/s)	160.2 (28.9)	110.1 (36.9)	**0.002**	1.990
	External rotation (deg/s)	114.1 (24.7)	104.4 (20.4)	0.327	0.470
	Internal rotation (deg/s)	68.8 (16.4)	76.9 (38.3)	0.531	0.343

Data are represented as means (SD) or medians (interquartile range). Bold texts indicate significant *p* values.

## Data Availability

The data used to support the findings of this study are available from the corresponding author upon request.
